# Morphological variations of the interatrial septum in ovine heart

**DOI:** 10.1371/journal.pone.0209604

**Published:** 2018-12-19

**Authors:** Mateusz K. Hołda, Agnieszka Pietsch-Fulbiszewska, Marek Trybus, Mateusz Koziej

**Affiliations:** 1 HEART—Heart Embryology and Anatomy Research Team, Department of Anatomy, Jagiellonian University Medical College, Cracow, Poland; 2 University Center of Veterinary Medicine, Jagiellonian University–University of Agriculture, Cracow, Poland; 3 2nd Department of General Surgery, Jagiellonian University Medical College, Krakow, Poland; York University, CANADA

## Abstract

Smooth septum interatrial septum, patent foramen ovale (PFO) channel and atrial septal pouches (SPs) are commonly described variants in humans. Recent discoveries on the clinical significance of left-sided SP may encourage the creation of new strategies and devices for the management of SPs. However, these strategies may first be tested in the ovine model before implementation in humans. Unfortunately, little is known about the presence of SPs in ovine. In this study a total of 60 ovine (*Ovis aries*) hearts were examined. The interatrial septum morphology was assessed and the PFO channel and SPs were measured. The most commonly occurring variant were PFO channels (25.0%) with channel lengths of 5.4±2.3 mm. Smooth septums were observed in 18.3% of hearts. In the remaining cases, septums had a left septal ridge (15.0%), left SP (11.7%), left septal bridge (10.0%), right SP (10.0%), or had both a right SP and left septal ridge (10.0%). No double SPs were observed. The mean right SP depth was 3.4 ± 1.2 mm, and its mean ostium width and height were 7.9±1.8 mm and 2.8±1.0, respectively. For the left SP, the mean depth was 6.0±1.7mm, the ostium width was 7.9±2.4mm, and the ostium height was 4.1±1.6mm (range: 2.3–6.4mm). In conclusion the interatrial septum of ovine hearts exhibit morphologies that are more similar to humans than they are to swine, which should be taken into account during experimental studies. The presence of a left SP in sheep hearts make ovine models a promising alternative to the human heart for developing left-sided SP management devices and techniques.

## Introduction

**Fig 1:**
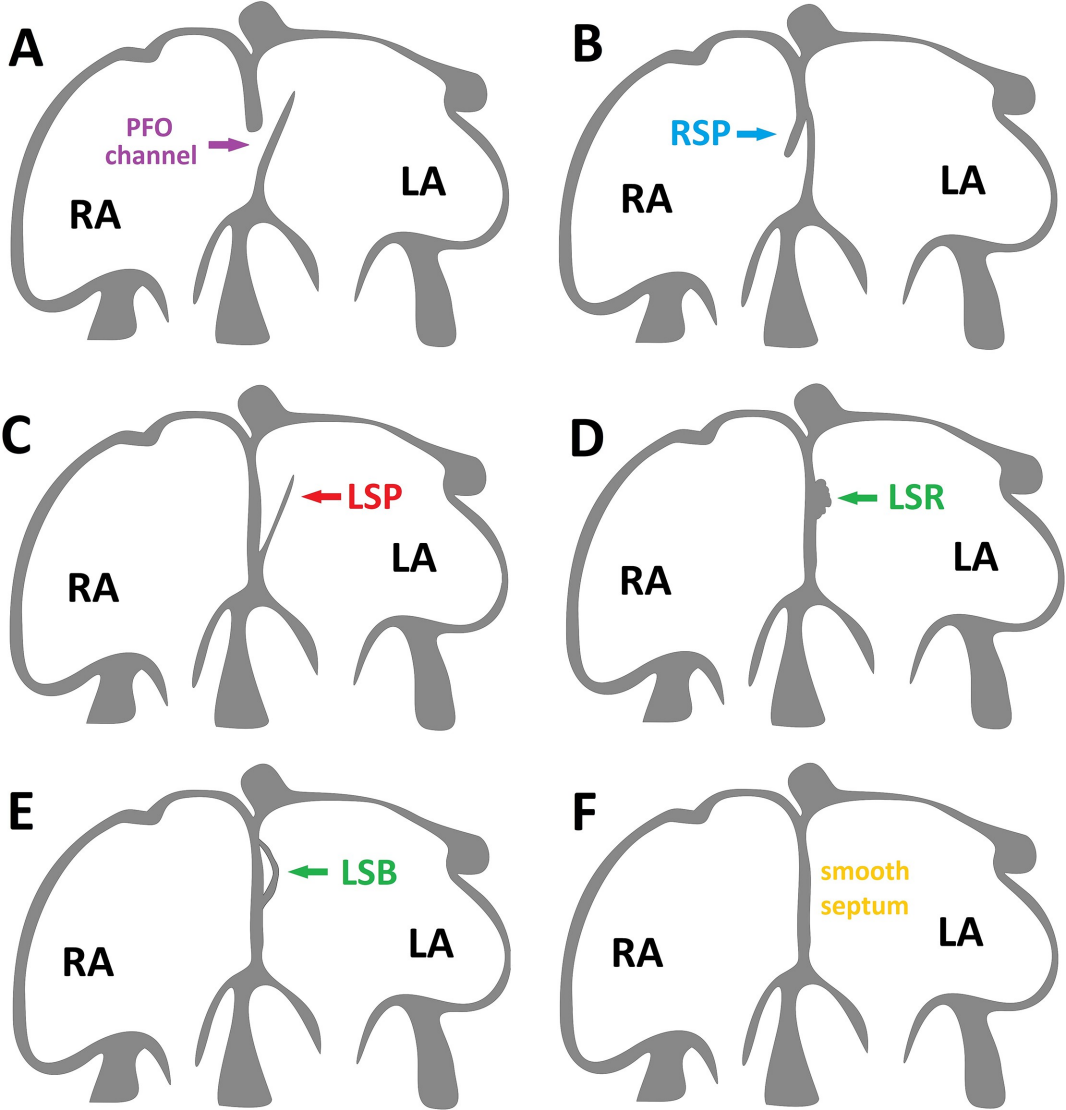
Schematic view of the interatrial septum in the ovine heart. [A] Patent foramen ovale (PFO) channel connecting both atria. [B] Right septal pouch (RSP)–diverticulum located on the right side of the interatrial septum, without connection between both atria across the septum (fusion limited to the superior portion zone of overlap). [C] Left septal pouch (LSP)–diverticulum located on the left side of the interatrial septum, without connection between both atria across the septum (fusion limited to the inferior portion of the zone of overlap). [D] Left septal ridge (LSR)–tissue fold on the left side of the interatrial septum in the location in which the LSP would be expected. [E] Left septal bridge (LSB)–tissue bridge located on the left side of the interatrial septum in the location in which the LSP would be expected. [F] Smooth septum—fusion between the septum primum and secundum occurs along the entire zone of overlap. LA–left atrium, RA–right atrium.

The interatrial septum located between right and left atrium shows great variability in humans. Smooth septum, patent foramen ovale (PFO) channel and atrial septal pouches (SPs) are commonly described variants [1]. The PFO channel is defined as a channel-like communication between the atria through an unfused septum (Fig 1A). The SP is a small, blind ended, kangaroo-like diverticulum that may be located on the left or/and right side of the interatrial septum, moreover there is no connection between the left and right atrium across the septum (Fig 1B and 1C) [2, 3]. Pouches are formed during postnatal life as a result of the incomplete fusion of the PFO channel, in other words constant friction between PFO channel and interatrial septum elements leads to gradually closure of channel and creation of SP [3]. All above mentioned morphological types of the interatrial septum should be considered as normal variants of the septum rather than as pathologies [3]. However, recent studies have proved that SP located on the left side of the septum may trigger atrial fibrillation and be a source of thrombosis, which significantly increases the risk of ischemic stroke [4–6].

Gross anatomy of large mammals hearts, such as dogs, pigs, sheep, and non-human primates share many similarities, but there are significant differences in the structure of the individual parts of their hearts [7–9]. The ovine heart is widely used as a research model for the human heart, especially in experimental models of mitral regurgitation and cardiac arrhythmias [10–12]. In addition, sheep hearts could be used for pre-clinical testing of cardio-vascular devices, developing new interventional and operational techniques, as well as for educational and training purposes [13]. Ovine interatrial septums are also useful as a model for the human septum [14–16].

Due to the recent discovery that left-sided SPs are clinically relevant, new strategies and devices for the management of SPs will need to be developed. However, these strategies will first need to be tested in the ovine model. Unfortunately, ovine interatrial septum anatomy is poorly discovered, and nothing is known about the presence of SPs in this species. Thus, in this study, we aimed to evaluate the morphology of ovine hearts interatrial septum and to compare it with the human and swine observations.

## Materials and methods

We examined 60 hearts from sheep (*Ovis aries*, Corriedale, <12 months old, ~37 kg). Hearts were dissected up to one hour after commercial animal slaughter. No animals were sacrificed deliberately for this study, and all samples were originally intended for use in the food industry. All aspects of the research complied to the legal requirements of the country in which the research was conducted. All specimens were free of congenital heart defects and were obtained from healthy animals that had no recognizable diseases. The hearts were obtained intact, washed free of blood and blood clots, weighed, and then fixed by immersion in 10% formaldehyde phosphate-buffered solution.

After a two-week fixation period, the right atrium was opened using an intercaval incision extending between orifices of the inferior and superior venae cava. The left atrium was opened from the posterior aspect using a transverse cut between the pulmonary veins ostia. Morphology of the interatrial septum was evaluated from the left and right atrial sides using the probe. Based on our previous studies, morphological variants of the interatrial septum morphology were classified as one of the following (Fig 1) [3, 17]:

- PFO channel–channel connecting the left and right atrium (Fig 1A);
- right SP–pouch opened into the right atrial cavity without a connection between the left and right atrium (Fig 1B);
- left SP–pouch opened into the left atrial cavity without a connection between the left and right atrium (Fig 1C);
- double SP–two pouches with openings into both atria without a connection between the left and right atrium;
- left septal ridge–the tissue fold on the left side of the interatrial septum in the location in which the left SP would be expected (Fig 1D);
- left septal bridge–tissue bridge located on the left side of the interatrial septum in the location in which the left SP would be expected (Fig 1E);
- smooth septum–no PFO channel, pouch, or other structures were observed (Fig 1F).

The length of the PFO channel, the SP depth, and its ostium width and height were measured as previously described [3]. Linear measurements were collected using 0.03 mm-precision electronic calipers (YATO, YT–7201, Poland). Two independent researchers performed measurements to minimize human bias. Measurements were repeated if there was a difference >10% for the same parameter. The mean of the two measurements was reported as the final result.

Data were presented as mean values with the corresponding standard deviation and range or as a percentage. The qualitative variables were compared using the χ2 test of proportions for categorical variables. Correlation coefficients were calculated to measure statistical dependence between the parameters of the measured hearts. We performed statistical analysis using StatSoft STATISTICA 13.1 software for Windows (StatSoft Inc., Tulsa, OK, USA). A p-value of 0.05 was considered statistically significant.

## Results

**Fig 2:**
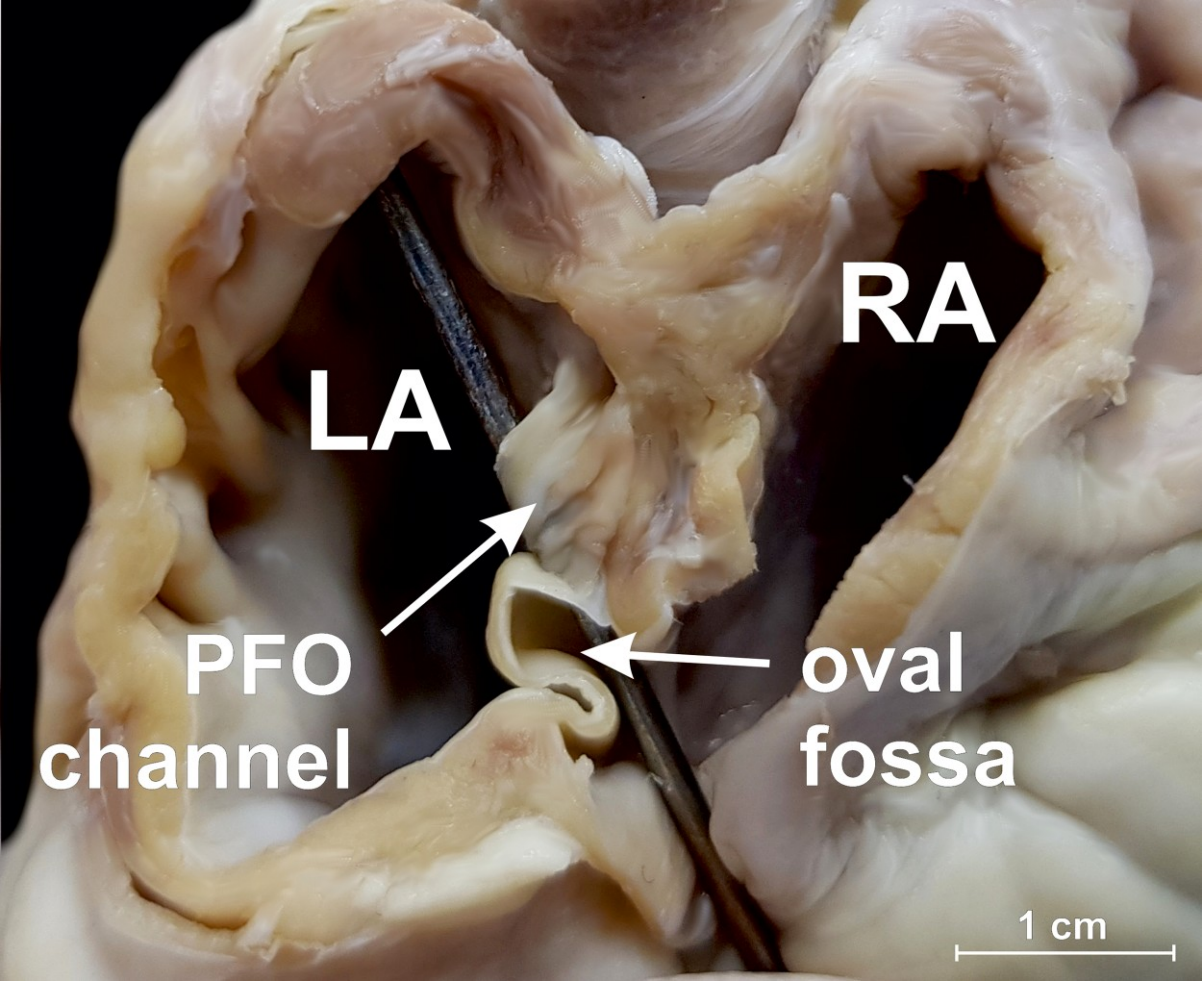
Photograph of ovine heart specimen showing the patent foramen ovale (PFO) channel. View from the back side, the interatrial septum and atrial walls were partially dissected, and the probe was inserted into the PFO channel. LA–left atrium, RA–right atrium.

**Fig 3:**
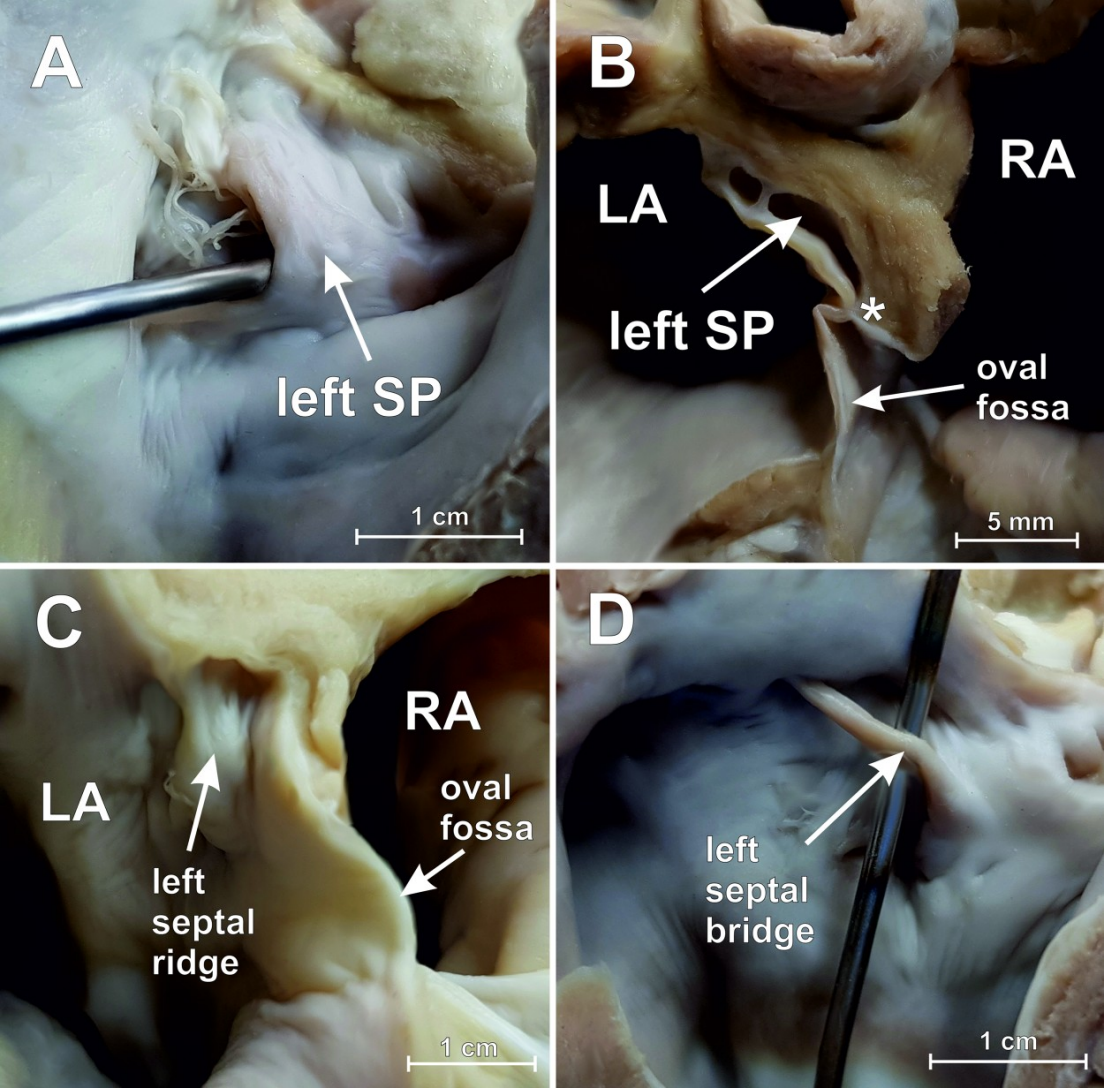
Photographs of ovine heart specimens showing different structures on the left side of the interatrial septum (view from the back side). [A] Left septal pouch (SP) with probe inserted into the pouch. [B] Section through the interatrial septum with the left SP; point of adhesion between septum primum and secundum could be seen (*). [C] Left septal ridge. [D] Left septal bridge. LA–left atrium, RA–right atrium.

**Fig 4:**
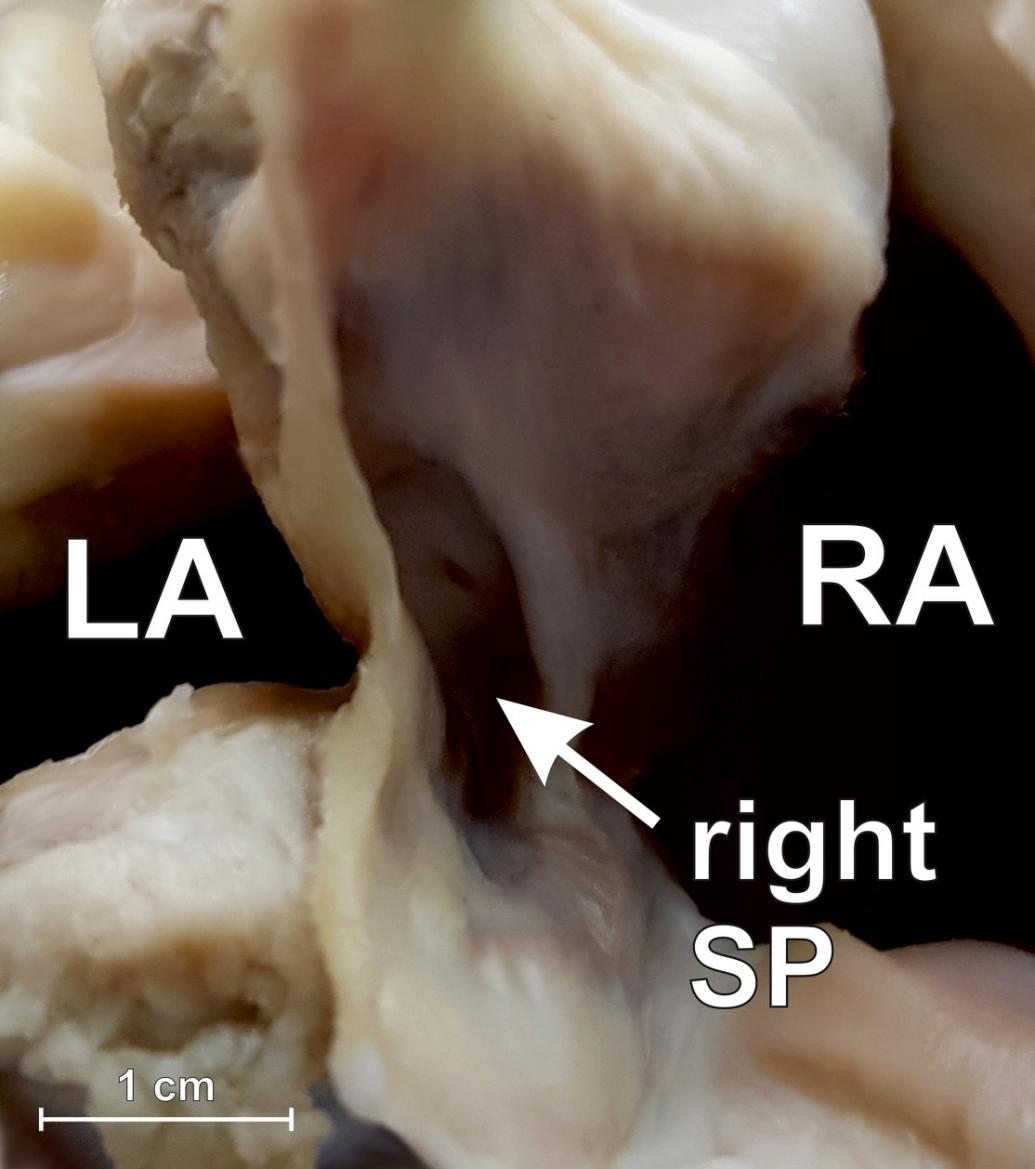
Photograph of porcine heart specimen showing section through the interatrial septum with the right septal pouch (SP) present (view from the back side). LA–left atrium, RA–right atrium.

The mean heart weight was 156.9 ± 36.6 g (range: 76.2 to 270.5 g). The most common variant of the interatrial septum was the PFO channel (25.0%) (Fig 2) with the mean channel length of 5.4 ± 2.3 mm (range: 2.3 to 10.4 mm). Smooth septum was observed in 18.3% of hearts. Left SP was observed in 11.7% of cases (Fig 3A and 3B), while the left septal ridge (Fig 3C) and left septal bridge (Fig 3D) were seen in 15.0% and 10.0%, respectively. The overall prevalence of right SP was 20.0% (Fig 4), but single right SPs were observed in 10.0% of all investigated cases and the other 10.0% included hearts with both a right SP and a left septal ridge. No double SPs were observed.

The mean right SP depth was 3.4 ± 1.2 mm (range: 1.8 to 6.4 mm), and its mean ostium width and height were 7.9 ± 1.8 mm (range: 5.6 to 10.6 mm) and 2.8 ± 1.0 (range: 1.6 to 5.4 mm), respectively. For the left SP, the mean depth was 6.0 ± 1.7 mm (range: 3.8 to 8.6 mm), ostium width was 7.9 ± 2.4 mm (range: 4.8 to 12.6 mm), and ostium height was 4.1 ± 1.6 mm (range: 2.3 to 6.4 mm).

Left SPs were significantly deeper (p = 0.001) and had larger ostium height (p = 0.04) than right SPs. There were no differences between the dimensions of the right SPs when the left septal ridge was present or absent (p>0.05). There were no correlations between any measured SPs dimensions and heart weight (p>0.05).

## Discussion

The fusion between the flap valve of the foramen ovale (septum primum) and the antero-superior rim of the oval fossa (septum secundum) may occur completely (smooth septum) or be limited only to one of three levels: superior, central, and inferior. Fusion limited to the superior portion of the zone of overlap leads to left SP, central to double SP, and inferior to right SP. When no fusion occurs, the PFO channel is visible. The remodeling of the interatrial septum starts postnatally and is lifelong [1, 3, 18].

Table 1 presents the comparison of morphological variations of interatrial septum of human, swine, and ovine hearts [2, 3, 17]. Three of compared studies assessing the interatrial septum configuration in different species were performed by our team, thus all possible differences resulting from the application of different criteria for identifying septal structures were minimized. Moreover, Table 1 included results of the study by Krishnan and Salazar, which investigated the morphology of the human interatrial septum, however the prevalence of double SPs, left septal ridges and bridges were not reported in this study and thus these results are not considered in further comparisons [2]. There are obvious differences in interatrial septum morphology between studied species. The most prominent one is concerning left SP prevalence, which is completely absent in swine and found in 11.7% of sheep and 41.5% of adult human hearts. The dimensions of ovine left SPs are smaller than human left SPs, however the differences are statistically insignificant (depth: 6.0 ± 1.7 vs. 8.4 ± 5.1 mm, p = 0.22; ostium height: 4.1 ± 1.6 vs. 5.2 ± 1.7 mm, p = 0.10; ostium width: 7.9 ± 2.4 vs. 8.8 ± 2.6 mm, p = 0.38) (Table 2). Therefore, the ovine heart may be used as a research model for left SP.

**Table 1:** Comparison of morphological variations of interatrial septum of human, swine and ovine hearts.

Structure	Human (n = 94) [2]	Human (n = 200) [3]	Swine (n = 75) [17]	Ovine (n = 60) *current study*
**PFO channel**	27.7%	25.0%	22.7%	25.0%
**Smooth septum**	28.7%	22.5%	26.6%	18.3%
**Right SP**	4.3%	5.5%	22.7%	10.0%
**Left SP**	39.4%	41.5%	0%	11.7%
**Double SP**	nr	5.5%	0%	0%
**Left septal ridge**	nr	0%	18.7%	15.0%
**Left septal bridge**	nr	nr	0%	10.0%
**Right SP + left septal ridge**	nr	0%	9.3%	10.0%

PFO–patent foramen ovale, SP–septal pouch, nr–not reported

**Table 2:** Comparison of interatrial septum structures measurements (mean ± standard deviation [mm]) performed in human, swine and ovine hearts.

	Human (n = 200) [3]	Swine (n = 75) [17]	Ovine (n = 60) *current study*	p-value ovine vs. human	p-value ovine vs. swine
**PFO channel length**	9.2 ± 3.9	7.1 ± 1.5	5.4 ± 2.3	0.02	<0.001
**left SP depth**	8.4 ± 5.1	-	6.0 ± 1.7	<0.001	-
**left SP ostium height**	5.2 ± 1.7	-	4.1 ± 1.6	<0.001	-
**left SP ostium width**	8.8 ± 2.6	-	7.9 ± 2.4	0.02	-
**right SP depth**	6.2 ± 3.4	6.3 ± 2.2	3.4 ± 1.2	<0.001	<0.001
**right SP ostium height**	4.5 ± 1.5	5.3 ± 1.6	2.8 ± 1.0	<0.001	<0.001
**right SP ostium width**	7.5 ± 3.5	5.8 ± 1.2	7.9 ± 1.8	0.4	<0.001

PFO–patent foramen ovale, SP—septal pouch

The presence of the PFO channel is comparable between ovine, swine, and human hearts. Nevertheless, there were differences in PFO channel length, which may have direct influence on the variations within the configuration of the interatrial septum. Ovine hearts have the shortest PFO channel when compared to swine (5.4 ± 2.3 vs. 7.1 ± 1.5 mm; p = 0.02) [17] and humans (5.4 ± 2.3 vs. 9.2 ± 3.9 mm; p<0.001) (Table 2) [3]. However, after standardization to heart weight the mean PFO channel length/heart weight ratio is the greatest in ovine heart (0.039 ± 0.018), followed by humans (0.025 ± 0.011) and swine (0.019 ± 0.010). Ovine hearts had the largest relative zone of overlap between PFO channel walls which may explain why we observed significantly more left-sided structures in sheep (left SP, ridge, and bridge) than in humans and swine. As such, we did not observe the presence of left SP in swine, which had relatively the shortest PFO channels [17].

The oval fossa in sheep is positioned more posteriorly, toward the inferior vena cava ostium and the septum secundum (infolding between the junction of the superior vena cava and right-sided pulmonary veins), and is significantly longer and more prominent than it is humans [8]. Three factors may be responsible for this arrangement. Firstly, the unguligrade posture of the sheep forces a different conformation of the ovine thorax, which is compressed in lateral manner, whereas dorsoventral compression is present in humans [19]. Secondly, different arrangement of the caval veins was observed between species. In ovine hearts, similar to swine, the inferior vena cava is rotated towards the left side and thus its main veins are not aligned along the same axis and are open into the right atrium at right angles to each other [20]. Moreover, the sheep heart has a small (10 x 2 x 2 mm) fully formed bone (*Os cordis*) located within the atrial septum at the point of attachment of the mitral valve anterior leaflet and lateral attachment of the aortic root [21]. All of this may have direct influence on the shift of the point of fusion between PFO channel walls and on the variation of interatrial septum components.

Since only one tenth of sheep (under 12-month of postnatal life) may have a left SP, its possible presence should be carefully evaluated. The left SP may be easily visualized using transesophageal echocardiography or contrast-enhanced ECG-gated multislice computed tomography of the heart [4, 22, 23]. As demonstrated previously, the prevalence of additional interatrial structures decreases in favor of the smooth septum as a function of age [3]. Therefore, the selection of animals of proper age is also important. Morphological studies using ovine heart specimens of different ages should be performed to establish the best animal age for use as left SP models.

## Conclusions

The interatrial septum of ovine hearts exhibit morphologies that are more similar to humans than they are to swine, which should be taken into account during experimental studies. The presence of a left SP in sheep hearts make ovine models a promising alternative to the human heart for developing left-sided SP management devices and techniques.

## Supporting information

S1 Table Dataset. (PDF)

## References

[1] Klimek-Piotrowska W, Hołda MK, Koziej M, Piątek K, Hołda J. Anatomy of the true interatrial septum for transseptal access to the left atrium. Annals of Anatomy, 2016;205:60–64. 10.1016/j.aanat.2016.01.009 26879344

[2] Krishnan SC, Salazar M. Septal pouch in the left atrium: a new anatomical entity with potential for embolic complications. *JACC Cardiovasc Interv*, 2010;3:98–104. 10.1016/j.jcin.2009.07.017 20129577

[3] Hołda M, Koziej M, Hołda J, Piątek K, Tyrak K, Chołopiak W, et al Atrial septal pouch–morphological features and clinical considerations. *International Journal of Cardiology*, 2016;220:337–342. 10.1016/j.ijcard.2016.06.141 27390952

[4] Hołda MK, Koziej M, Wszołek K, Pawlik W, Krawczyk-Ożóg A, Sorysz D, et al Left atrial accessory appendages, diverticula, and left-sided septal pouch in multi-slice computed tomography. Association with atrial fibrillation and cerebrovascular accidents. *International Journal of Cardiology*, 2017;244:163–168. 10.1016/j.ijcard.2017.06.042 28629626

[5] Sun JP, Meng F, Yang XS, Lee AP, Chen M, Zhang B, et al Prevalence of atrial septal pouch and risk of ischemic stroke. *International Journal of Cardiology*, 2016;214:37–40. 10.1016/j.ijcard.2016.03.119 27057971

[6] Hołda M, Krawczyk-Ożóg A, Koziej M, Sorysz D, Hołda J, Dudek D, et al Left-sided atrial septal pouch is a risk factor for cryptogenic stroke. *J Am Soc Echocardiogr*, 2018;31:771–776. 10.1016/j.echo.2018.01.023 29573928

[7] Sands MP, Rittenhouse EA, Mohri H, Merendino KA. An anatomical comparison of human pig, calf, and sheep aortic valves. *Ann Thorac Surg*, 1969;8:407–14. 5353458 10.1016/s0003-4975(10)66071-7

[8] Michaëlsson M, Ho SY. Congenital heart malformations in mammals: an illustrated text Imperial College Press, 2000.

[9] Camacho P, Fan H, Liu Z, He JQ. Large Mammalian Animal Models of Heart Disease. *J Cardiovasc Dev Dis*, 2016;3:30. 10.3390/jcdd3040030 PMC5715721 29367573

[10] Timek TA, Lai DT, Tibayan F, Daughters GT, Liang D, Dagum P, et al Atrial contraction and mitral annular dynamics during acute left atrial and ventricular ischemia in sheep. *Am J Physiol Circ Physiol*, 2002;283:H1929–H1935. 10.1152/ajpheart.00149.2002 12384471

[11] Hill AJ, Iaizzo PA. Comparative cardiac anatomy. In: Handbook of Cardiac Anatomy, Physiology, and Devices, *Third Edition*, 2015:89–114.

[12] Butters TD, Aslanidi O V, Zhao J, Smaill B3, Zhang H. A novel computational sheep atria model for the study of atrial fibrillation. Interface Focus, 2013;3:20120067 10.1098/rsfs.2012.0067 24427521 PMC3638473

[13] DiVincenti L, Westcott R, Lee C. Sheep (Ovis aries) as a model for cardiovascular surgery and management before, during, and after cardiopulmonary bypass. *J Am Assoc Lab Anim Sci*, 2014;53:439–48. 25255065 PMC4181684

[14] Jux C, Bertram H, Wohlsein P, Bruegmann M, Paul T. Interventional Atrial Septal Defect Closure Using a Totally Bioresorbable Occluder Matrix. Development and Preclinical Evaluation of the BioSTAR Device. *J Am Coll Cardiol*, 2006;48:161–169. 10.1016/j.jacc.2006.02.057 16814662

[15] Sigler M, Jux C. Biocompatibility of septal defect closure devices. *Heart*, 2007;93:444–449. 10.1136/hrt.2006.098103 17035510 PMC1861502

[16] Uchida BT, Pavcnik D, Shimohira M, Choi YH, Jeromel M, Keller FS. New coaxial transseptal needle for creation of atrial septal defects in adult sheep. *Cardiovasc Intervent Radiol*, 2011;34:620–625. 10.1007/s00270-010-9948-1 20661566

[17] Hołda MK, Hołda J, Koziej M, Piątek K, Klimek-Piotrowska W. Porcine heart interatrial septum anatomy. *Annals of Anatomy*, 2018;217:24–28. 10.1016/j.aanat.2018.01.002 29458135

[18] Anderson RH, Brown NA, Webb S. Development and structure of the atrial septum. *Heart*, 2002;88:104–110. 12067964 10.1136/heart.88.1.104 PMC1767197

[19] Lelovas PP, Kostomitsopoulos NG, Xanthos TT. A comparative anatomic and physiologic overview of the porcine heart. *J Am Assoc Lab Anim Sci*, 2014;53:432–438. 25255064 PMC4181683

[20] Dawes GS, Mott JC, Widdicombe JG. The foetal circulation in the lamb. *J Physiol*, 1954;126:563–587. 13222355 10.1113/jphysiol.1954.sp005227 PMC1365878

[21] Frink RJ, Merrick B. The sheep heart: Coronary and conduction system anatomy with special reference to the presence of an os cordis. *Anat Rec*, 1974;179:189–199. 10.1002/ar.1091790204 4829081

[22] Hołda MK, Krawczyk-Ożóg A, Koziej M, Pawlik W, Sorysz D, Wszołek K, et al Cardiac computed tomography compared with two-dimensional transesophageal echocardiography for the detection and assessment of atrial septal pouches. *Int J Cardiovasc Imaging*, 2018;34:1305–1313. 10.1007/s10554-018-1342-0 29574626

[23] Hołda M, Krawczyk-Ożóg, Agata Koziej M, Sorysz D, Hołda J, Dudek D, et al Mid-esophageal bicaval versus short-axis view of interatrial septum in two-dimensional transesophageal echocardiography for diagnosis and measurement of atrial septal pouches. Echocardiography, 2018;35:827–833. 10.1111/echo.13847 29490109

