# Supplementary material for: Morphological variations of the interatrial septum in ovine heart
Source: PLoS One. 2018 Dec 19;13(12):e0209604. doi: 10.1371/journal.pone.0209604 (PMC6300291; doi:10.1371/journal.pone.0209604)
Supplement: S1 Table — (PDF) [file pone.0209604.s001.pdf]

No	heart weight	IAS	PFO length	d RSP	w RSP	h RSP	d LSP	wć LSP	h LSP
1	140	4		3,7	3,5	10,6			
2	117	2	7,7						
3	146	1							
4	267	6							
5	175	8		3,1	3,6	10,1			
6	157	2	4,7						
7	148	2	2,3						
8	189	8		1,8	2,4	5,6			
9	165	2	3,5						
10	163	5							
11	91	5							
12	176	3					4,1	2,6	6,3
13	155	5							
14	144	2	5,1						
15	148	8		4,8	5,4	9,1			
16	162	1							
17	197	5							
18	138	6							
19	162	1							
20	141	1							
21	131	4		2,8	2,9	6,9			
22	113	6							
23	156	3					3,8	2,3	4,8
24	145	4		3,1	1,9	8,8			
25	169	1							
26	130	2	4,7						
27	205	4		3,4	2,3	6,2			
28	117	5							
29	186	4		6,4	3,1	6,6			
30	148	6							
31	120	6							
32	268	2	9,9						
33	156	1							
34	143	2	10,4						
35	161	8		3,7	1,8	10,1			
36	184	2	5,5						
37	126	2	4,3						
38	270	3					6,1	3,2	7,8
39	190	1							
40	147	5							
41	153	5							
42	76	2	4,9						
43	134	2	3,8						
44	121	2	6,7						
45	180	3					8,6	4,2	7,7
46	147	3					6,2	3,8	7,3
47	153	2	3,1						
48	191	1							
49	170	5							
50	202	8		2,1	1,6	5,7			
51	155	5							
52	122	4		2,9	2,4	7,5			
53	107	8		3,1	2,6	7,5			
54	167	1							
55	138	2	4,4						
56	162	3					6,2	6,4	8,8
57	147	6							
58	121	3					7,1	6,2	12,6
59	149	1							
60	172	1							
